# A Low-Cost Microfluidic Chip for Rapid Genotyping of Malaria-Transmitting Mosquitoes

**DOI:** 10.1371/journal.pone.0042222

**Published:** 2012-08-03

**Authors:** Changchun Liu, Michael G. Mauk, Robert Hart, Mariangela Bonizzoni, Guiyun Yan, Haim H. Bau

**Affiliations:** 1 Department of Mechanical Engineering and Applied Mechanics, University of Pennsylvania, Philadelphia, Pennsylvania, United States of America; 2 College of Health Sciences, University of California Irvine, Irvine, California, United States of America; Kansas State University, United States of America

## Abstract

**Background:**

Vector control is one of the most effective measures to prevent the transmission of malaria, a disease that causes over 600,000 deaths annually. Around 30–40 *Anopheles* mosquito species are natural vectors of malaria parasites. Some of these species cannot be morphologically distinguished, but have behavioral and ecological differences. Emblematic of this is the *Anopheles gambiae* species complex. The correct identification of vector species is fundamental to the development of control strategies and epidemiological studies of disease transmission.

**Methodology/Principal Findings:**

An inexpensive, disposable, field-deployable, sample-to-answer, microfluidic chip was designed, constructed, and tested for rapid molecular identification of *Anopheles gambiae* and *Anopheles arabiensis*. The chip contains three isothermal amplification reactors. One test reactor operates with specific primers to amplify *Anopheles gambiae* DNA, another with specific primers for *Anopheles arabiensis* DNA, and the third serves as a negative control. A mosquito leg was crushed on an isolation membrane. Two discs, laden with mosquito tissue, were punched out of the membrane and inserted into the two test chambers. The isolated, disc-bound DNA served as a template in the amplification processes. The amplification products were detected with intercalating fluorescent dye that was excited with a blue light-emitting diode. The emitted light was observed by eye and recorded with a cell-phone camera. When the target consisted of *Anopheles gambiae*, the reactor containing primers specific to *An. gambiae* lit up while the other two reactors remained dark. When the target consisted of *Anopheles arabiensis*, the reactor containing primers specific to *An. arabiensis* lit up while the other two reactors remained dark.

**Conclusions/Significance:**

The microfluidic chip provides a means to identify mosquito type through molecular analysis. It is suitable for field work, allowing one to track the geographical distribution of mosquito populations and community structure alterations due to environmental changes and malaria intervention measures.

## Introduction

Malaria is one of the most prevalent and burdensome infectious diseases in the world today, especially in tropical and subtropical regions, and continues to be a major global health problem, with over 40% of the world’s population exposed to varying degrees of malaria risk. It is estimated that over 500 million people suffer from malaria infections annually, resulting in more than 600,000 deaths [1]–[4]. A major breakthrough in understanding malaria was made about one hundred years ago by Sir Ronald Ross, who first demonstrated that the malarial parasites, *Plasmodium* species, were transmitted by mosquitoes [5]. Since Ross’s discovery, one of the most successful methods of malaria prevention and eradication has been through control of the mosquito vector. All of the *Plasmodium* species that cause human malaria are transmitted by mosquitoes of the genus *Anopheles*. Of the approximately 430 *Anopheles* species, about 30–40 are vectors of malaria parasites [6]. Some malaria vector species can be morphologically indistinguishable, but have ecological and behavioral differences. For example, *Anopheles gambiae sensu latu* (s.l.) complex consists of seven morphologically indistinguishable mosquito species, including *Anopheles gambiae sensu strictu* (hereafter referred to as *An. gambiae*) and *An. arabiensis*, the main malaria vectors in sub-Saharan Africa [7]–[10]. Although *An. gambiae* and *An. arabiensis* are often sympatric, they differ greatly in their ability to vector malaria parasites, blood-feeding host preferences, resistance to desiccation, larval habitat requirements, and responses to the application of insecticide-treated bed nets (ITNs) [11], [12]. Assessment of malaria risks, deployment of vector control techniques, and evaluation of the impact of control measures would benefit from knowledge of the identity, spatial distribution, and abundance of the various vector species. The development of simple, rapid, low-cost, and reliable tools for mosquito species identification in the field may lead to a better understanding of intraspecies genetic diversity and population structure and may play an important role in the development of effective vector control strategies [11], [13].

Various methods have been developed to identify individual *An. gambiae* species. See Collins and Paskewitz [14] for a lucid review. Briefly, in the past, the identification of the *An. gambiae* species complex has been achieved mostly by using polytene chromosome binding patterns [15], isoenzyme electrophoresis [16], and high-performance liquid chromatography of cuticular hydrocarbons [17]. All these techniques are laborious, require highly skilled personnel, are applicable only to certain cells, and require laboratory facilities. With the advent of Polymerase Chain Reaction (PCR) technology, the molecular approach has greatly improved the accuracy of mosquito species identification [14], [18]–[20]. Genomic, DNA-based molecular methods of species identification are advantageous as they can be applied to specimens and situations unsuitable for morphological taxonomy. However, PCR-based molecular identification methods require relatively expensive and sophisticated laboratory equipment unavailable to public health practitioners in many developing countries.

Recently, loop mediated isothermal amplification (LAMP) technology was adapted for molecular discrimination between *An. gambiae* and *An. arabiensis* mosquito species, showing a sensitivity better than 0.9 and a 100% specificity compared with standard rDNA-PCR [11], [18] when testing field-captured mosquitoes. The use of isothermal amplification (instead of the thermal cycling needed for PCR) dramatically simplifies the molecular identification process, greatly simplifies the instrumentation needed [21], [22], and even allows instrument-free operation [23], [24].

In recent years, there have been considerable efforts to integrate biochemical analysis and medical diagnostics processes into monolithic microfluidic platforms [25], [26]. Compared to conventional laboratory methods, such integrated microfluidic implementations offer the advantages of low cost, short test times, small sample sizes, low reagent consumption, and most importantly, full automation of all processes from sample preparation to detection in a single device. The “sample-to-answer” capability is particularly attractive for resource-poor regions, where funds and trained personnel are in short supply [26]. Although a number of groups are developing microfluidic components for nucleic acid testing (NAT), there are, to date, only a few reports of fully-integrated, microfluidic NAT chips that can perform all the necessary steps from sample introduction and preparation to target detection [26], [27]. On-chip sample preparation (i.e., lysis, nucleic acid isolation, purification, and concentration) is still a challenge [26]–[30]. In an effort to simplify the design and operation of a microfluidic diagnostics system, our group has recently developed a multi-function, isothermal amplification reactor with an embedded isolation membrane, such as Flinders Technology Associates Whatman FTA®, without a need for nucleic acid elution [31]. The porous cellulose FTA membrane serves as a solid-phase binding medium for extraction, concentration, and purification of nucleic acids from cell lysates. We have used our devices to detect the presence of the HIV virus in saliva specimens. Our chip with an embedded FTA membrane successfully isolated viral RNA and carried out real-time, reverse-transcription, loop-mediated isothermal amplification (LAMP) with a detection limit better than ten target particles per sample [31].

In this paper, we will show that a similar idea can be used to distinguish mosquito species. Briefly, we report on a simple, low-cost, disposable, sample-to-answer, microfluidic chip, which integrates the functional steps of lysis of mosquito tissue cells; nucleic acid capture, concentration, and purification; isothermal amplification; and detection into a single chamber formed in a plastic substrate. A cell phone (iPhone™ 4) with an embedded CCD camera monitors the fluorescence signal emitted during the enzymatic amplification reaction in the microfluidic chip. We use a small piece of Whatman FTA filter paper for sample collection and nucleic acid isolation. The utility of our system was demonstrated by identifying the malaria-transmitting mosquitoes *An. gambiae* and *An. arabiensis*. To the best of our knowledge, this is the first report describing an integrated microfluidic chip for molecular identification of insect disease vectors with a cell phone recorder. The chip design and operation, including cell phone imaging for detection, can readily accommodate multiplexed analysis for parallel detection of several mosquito species and appropriate control reactions and also provides global positioning of the mosquito’s capture location.

## Materials and Methods

### Materials

The DNeasy™ Blood and Tissue kit, which includes AL (lysis and binding) buffer and AW1 and AW2 ethanol-based wash buffers, was purchased from Qiagen Inc. (Valencia, CA). The Loopamp™ DNA amplification kit was obtained from Eiken Chemical Co. Ltd. (Tochigi, Japan). SYTO-9 Green DNA binding dye was obtained from Invitrogen Corp. (Carlsbad, CA). Acetonitrile, ethanol, and Tris-acetate EDTA (TAE) buffer (10×) were purchased from Sigma Aldrich and used without further purification. The FTA card was obtained from Whatman (Florham Park, NJ). A 0.118 inch thick Polymethyl methacrylate (PMMA) sheet and a 0.01 inch thick, PMMA film were, respectively, supplied by McMaster-Carr and Cyro Industries. PCR Sealers™ tape (Microseal® ‘B’ Film) was purchased from Bio-Rad Laboratories (Hercules, CA).

*Anopheles gambiae* of the G3 strain and *An. Arabiensis* of the KGB strain were used in this study. Mosquitoes were reared at the insectary of the University of California at Irvine at 27°C with 77% humidity and a 12 hr day/night, 30 min dusk/dawn lighting cycle. One to two days old, non-bloodfed mosquitoes were stored individually in Ethanol 70% and shipped to the University of Pennsylvania.

The on-chip LAMP amplification used the LAMP primers previously described [11]. The LAMP master reaction mixture contained 20 mM Tris-HCl (pH 8.8), 10 mM KCl, 10 mM (NH_2_)SO_4_, 8 mM MgSO_4_, 0.1% Tween 20, 0.8 M betaine, 8U Bst DNA polymerase, 1.4 mM dNTPs, and 4.0 µM SYTO® 9 Green intercalating dye.

### Integrated Microfluidic Chip for Rapid Genotyping of Mosquitoes

**Figure 1:**
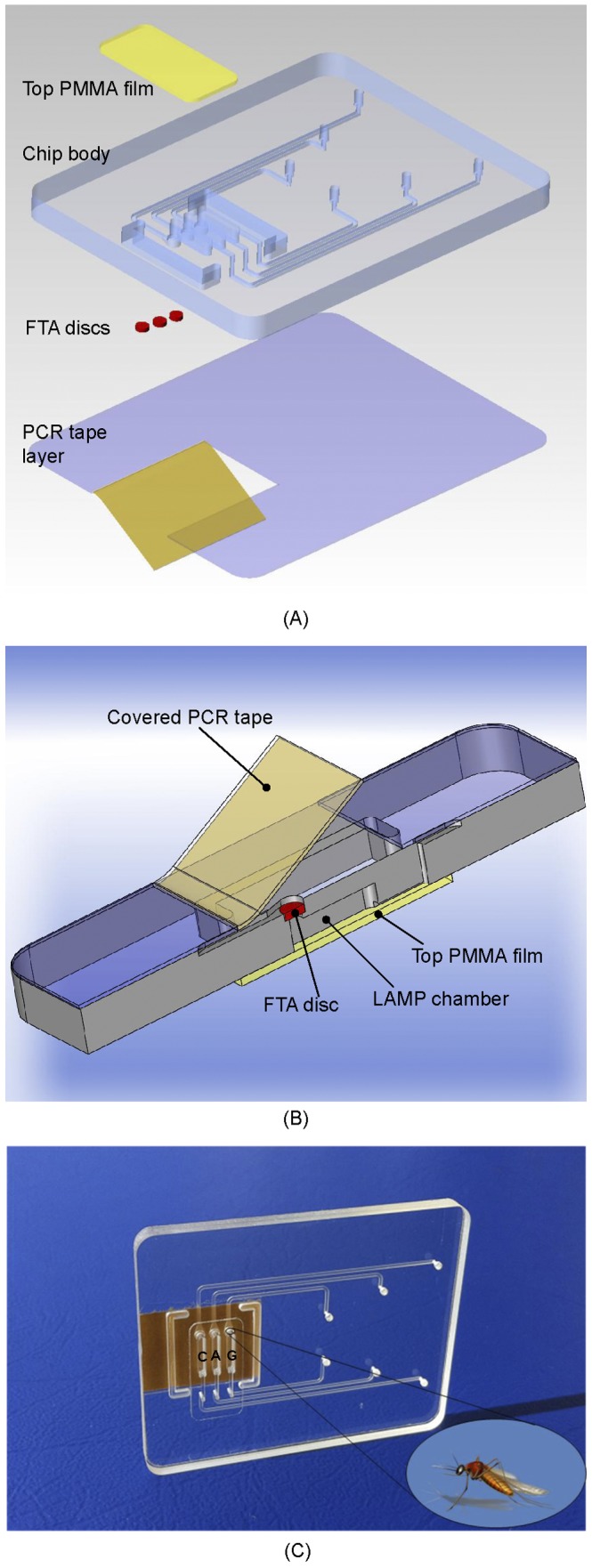
Microfluidic Chip housing three amplification chambers. (A) An exploded view of the chip used for genotyping of malaria-transmitting mosquitoes. The chip consists of three layers: a top PMMA film; a PMMA chip body, and a PCR Sealers™ tape bottom. The various features of the chip body were milled with a CNC machine. (B) Cross-sectional view of the assembled, inverted chip loaded with a FTA disc. In the test chambers, the disks carry pieces of a mosquito leg. (C) A photograph of the chip.

The chip designed for rapid genotyping of mosquitoes is shown in Figure 1. Figure 1A is an exploded view of the chip. The 46 mm×36 mm×3.50 mm chip consists of three layers: a top layer made with 250 µm (0.01 inch) thick Polymethyl methacrylate (PMMA) film; a 3 mm (0.118 inch) thick PMMA chip body, and a 250 µm (0.01 inch) thick PCR Sealers™ tape bottom. Both the top PMMA film and the PCR Sealers™ tape bottom were cut with a CO_2_ laser (Universal Laser Systems). The chip body was milled with a precision, computer-controlled (CNC) milling machine (HAAS Automation Inc.) to form three separate reactors (more are possible, if desired), FTA disc supports, and access conduits [31]–[33]. The top PMMA film was solvent-bonded with acetonitrile at room temperature. Residual solvent was removed by overnight heating at 50°C.

Figure 1B depicts schematically a cross-sectional view of an inverted (top side down) reactor with the installed FTA disc. The reactor is 5.2 mm in length, 1.0 mm in width, and 3.0 mm in depth. The total volume of the reactor is ∼16 µL. Each reactor is connected to separate inlet and exit ports with 500 µm wide×200 µm deep conduits. Figure 1C shows a photograph of the plastic chip containing three reactors. Two of the reactors are used as test reactors and are labeled “**G**” and “**A**”. The third reactor is used as a negative (no-target) control reactor and is labeled “**C**”. The reactors are surrounded by air-filled, closed trenches that act as thermal guards to minimize heat interactions between the reactors and the rest of the chip.

To carry out mosquito identification, FTA discs (see below) were inserted in the various reactors. The FTA disks operated in filtration mode. In other words, all liquids transmitted through the reactors passed through the FTA membrane. Once all the FTA disks had been secured in place, the reactors were sealed with adhesive cover (PCR Sealers™).

### Procedure

In our experiments, we used a Whatman FTA® card for nucleic acid isolation. FTA paper has been widely used to collect, store, purify, and transport genetic materials from a wide range of biological sources such as whole blood, buccal scrapes, tissues, plasmids, plants, and microorganisms [34]–[36].

**Figure 2:**
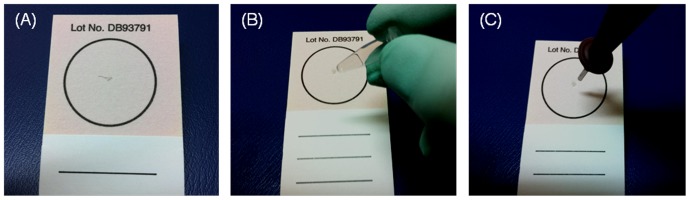
The sample preparation process. (A) A mosquito leg was placed on the FTA® card. (B) The leg was crushed with a blunt object. Witness the resulting gray spot on the FTA® card. (C) A 1.5-mm diameter FTA disc was punched out from the spotted area with a Harris punch cutter.

Various mosquito body parts can be used as the source of genetic material. We found the mosquito leg to be convenient to handle. A single mosquito leg was severed with tweezers and placed on the FTA® card (Figure 2A). Then, the leg was crushed with a blunt object (Figure 2B), which resulted in a gray spot on the FTA® card (Figure 2B). Next, a 1.5-mm diameter disk was punched out from the stained area with a Harris punch cutter (American MasterTech Scientific, Inc., Lodi, CA) (Figure 2C). The disk was then placed in the FTA disk holder of test reactor **G** (Figure 1B). The process was repeated and a second disk was cut from the stained area and placed in test reactor **A**. A blank disk was placed in the third reactor **C**, which serves as a negative (no template) control. Finally, the protective paper was removed from the adhesive side of the PCR Sealers™ tape, and the tape was attached to the chip’s body and sealed all three reactors (Figure 1B).

To effectuate nucleic acids isolation, 150 µl Qiagen AL buffer (an aqueous 6 M solution of chaotropic salt guanidinium HCl) was pipetted through the inlet ports into each of the three reactors. The AL buffer is a strong lysing agent that also promotes binding of nucleic acids to the Whatman FTA material. The sample was allowed to incubate with the lysis buffer for 3 minutes at room temperature. Since the sample contains a very large number of cells, some of which may lyse during the mechanical grinding (Figure 2B), and since the LAMP process is highly efficient, no attempt was made to optimize the incubation time.

The ledges formed in the chip to accommodate the FTA membrane acted as stoppers, keeping the membrane in place during the lysis/binding buffer flow. During this flow process, the wetted membrane expanded and sealed against the conduit wall, preventing any liquid from bypassing the membrane. To remove the AL buffer and any remaining potential inhibitors of enzymatic amplification, the FTA membrane was washed twice with ethanol-based buffers. In the first wash step, 150 µl of Qiagen AW1 buffer was pipetted through each reactor. In the second wash step, 200 µl of Qiagen AW2 buffer was pipetted through each reactor. The wash steps were followed with air-drying for 30 seconds. Next, various 20 µl of LAMP master mixes, which contained all the reagents necessary for the LAMP reaction along with 4.0 µM fluorescent DNA intercalating dye (SYTO® 9 Green), were injected into the three reactors through their inlet ports. LAMP reaction mix containing primers for *An. gambiae* was introduced into the first test reactor, dubbed reactor **G**. LAMP reaction mix containing primers for *An. arabiensis* was introduced into the second reactor, dubbed reactor **A**. LAMP reaction mix containing either primers for *An.gambiae, An. arabiensis,* or a mixture of the two was introduced into the third reactor, dubbed reactor **C** (Figure 1C). It is envisioned that in field applications, the reaction mixes will be pre-stored in the chip.

Once the various reaction mixes were inserted into the chip, the inlet ports and outlet ports were sealed with transparent tape (Scotch tape™, 3 M, St. Paul, MN) to minimize evaporation during the amplification process. Then, the reactors were heated to 63°C to facilitate the enzymatic amplification process.

In contrast to standard sample preparation procedures for enzymatic amplification, we did not elute the nucleic acids from the isolation membrane. Avoidance of the elution step greatly simplifies chip operation and flow control. In our application, the FTA filter paper served both as a solid-binding phase to isolate, concentrate, and purify the nucleic acids extracted from the mosquito cells and as a means to immobilize the template for the subsequent amplification reaction.

### The Amplification Process

**Figure 3:**
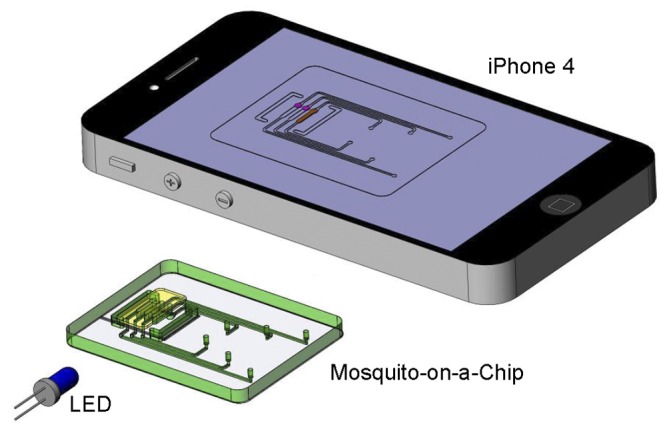
A schematic illustration of the experimental set-up for LAMP amplification and end-point detection. The chip was heated by a thin-film heater to maintain the reactors at 63±0.5°C. At the conclusion of the LAMP amplification process, the chip was illuminated from the side with a blue LED and the photo of the fluorescent image was taken with a cell phone camera (iPhone 4).

The experimental setup for on-chip LAMP amplification and end-point fluorescent detection is depicted in Figure 3A. Briefly, the system consists of a chip support equipped with a flexible, polyimide-based, thin film heater (HK5572R7.5L23A, Minco Products, Inc., Minneapolis, MN) and a type **T** thermocouple (Omega Engr., each wire 75 µm in diameter, and a junction diameter of ∼170 µm). The thermocouple junction was placed at the interface between the heater and the chip [21], [31]. The chip, once filled with the LAMP master mix, was fixed to the chip support with a double-sided adhesive tape, allowing the reactors to form a good thermal contact with the thin film heater. The heater was powered by a DC power supply (Model 1611, B&K Precision Corporation, CA). The power supply was adjusted to maintain the reactors at 63±0.5°C. Although the LAMP process is fairly forgiving to temperature variations, in field applications, it would be necessary to use a closed-loop thermal controller to accommodate operation over the broad range of ambient temperatures that may be encountered in various regions and times. An appropriate, custom-made thermal controller was previously described and can be used in this application [37]. Alternatively, one can use a *self-heating* chip, wherein the heat is generated by an exothermic reaction and the temperature is controlled with a phase change material [23]. The reactors were incubated for nearly an hour.

During the device development process, we found it useful to monitor the amplification process in real time. This was done by mounting a minute, portable reader on top of the chip as previously described [21], [31]. The real time measurements allowed us to determine the time needed for the reaction.

For the mosquito identification, we need only end-point detection. To this end, 60 minutes after the start of the heating, the chip was illuminated from its side with a small, blue LED light (Newark/Element14, IL, $0.19 per piece) with an approximate excitation wavelength of 470 nm. An image of the excited amplification reactors was also taken with a cell-phone camera (Apple iPhone 4). The reactors that contained amplification products were clearly visible as they emitted green light while reactors without amplification products remained dim. The cell phone camera provided a means to record the test results, to transmit test results to a central data processing site, and to record the geographic location (GPS) of the test.

To further confirm the amplification results, 5 µL of each LAMP-amplified product were removed from the three reactors with a pipette and subjected to gel electrophoresis in a 2.0% agarose gel. Electrophoresis of the amplified DNA was carried out in TAE (Tris-Acetate-EDTA) buffer at a constant voltage of 114 V for 40 minutes. DNA marker VIII (Roche Diagnostic, Indianapolis, Indiana, USA) was used to calibrate the size of the amplified DNA molecules in the various bands. The gel was stained with ethidium bromide and was visualized with a UV gel reader.

## Results and Discussion

### Real Time Detection

**Figure 4:**
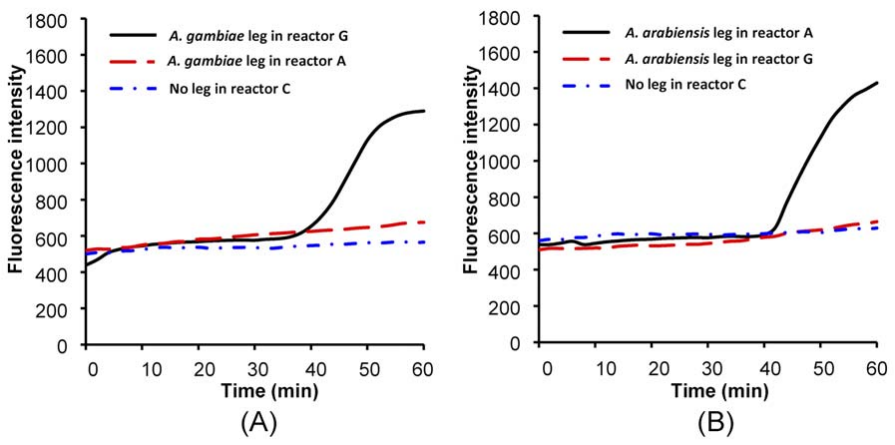
Real-time fluorescence amplification curves. Real-time monitoring of LAMP amplification products in reactors G, A, and C as functions of time when the sample consists of *An. gambiae* (A) and *An. arabiensis* (B).

Although, in this application, we need only a qualitative yes/no determination, it is still useful to monitor the amplification process in real time. Figure 4A depicts the signal intensity (in arbitrary units) emitted from amplification reactors **G**, **A**, and **C** as functions of time when the sample consisted of *An. gambiae.* Witness that there are no signals emitted from reactors **A** and **C**. No amplification occurs in reactors **A** and **C** since reactor **A** does not contain the appropriate primers and reactor **C** lacks the target. After a time delay of about 39 minutes, the signal from reactor **G** ramped up and eventually saturated, indicating that the primers in reactor **G** are compatible with the *An. gambiae* DNA.

Figure 4B depicts the signal intensity (in arbitrary units) emitted from amplification reactors **G**, **A**, and **C** as functions of time when the sample consisted of *An. arabiensis.* In this case, the signals emitted by reactors **G** (incompatible primers) and **C** (no target) remained flat, and the signal emitted from reactor **A** ramped up after a delay of about 43 minutes. Since in our application we are only interested in a yes/no answer, it suffices to detect the signal at one instant in time. Figure 4 suggests that a waiting period of 50–60 minutes would be adequate.

### End-point Detection

**Figure 5:**
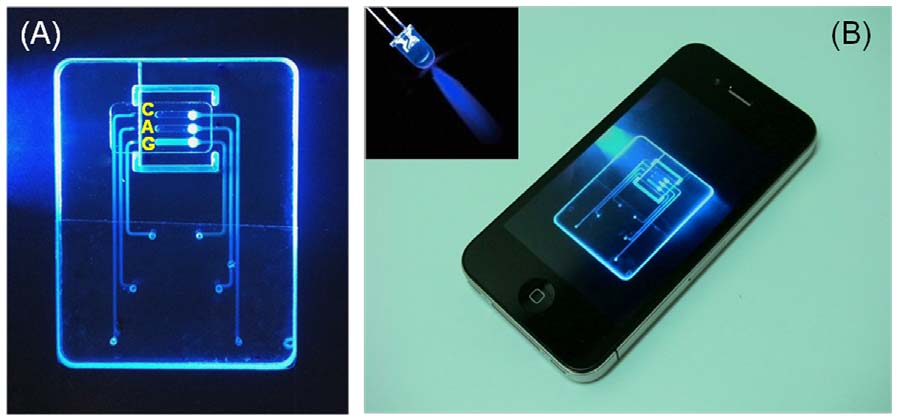
End-point detection of *An. gambiae* DNA recorded with iPhone 4. (A) An enlarged image of the illuminated chip. Reactor G emits visible light while reactors A and C stay dark. (B) Photograph of iPhone 4 with mosquito-on-a-chip fluorescence image. A minute, reusable, light-emitting diode (inset) excites the fluorescence emission.

An inexpensive blue LED was used to excite the intercalating dye that was included with the reaction mix. Figure 5A is a photograph of a chip containing a sample of *An. gambiae*. The light emitted from reactor **G** is clearly visible to the naked eye, indicating successful amplification of the *An. gambiae* DNA. Reactor **A** does not emit light, indicating the absence of *An. arabiensis* DNA. Similarly, the control reactor **C** did not emit any light, indicating that the reagents were not contaminated. Figure 5 illustrates that the device can operate without a need for a reader. Reader-free operation would allow significant cost savings.

The emission from the amplicons was captured with an Apple iPhone 4 camera with a resolution of 960×640 pixels per frame (Figure 5B). Witness that the image of the chip is clearly visible on the phone’s screen. Appropriate image analysis software could readily determine which reactor produces a positive signal and which reactor is negative. Moreover, the test results can be transmitted *via* the cell phone to a data processing center together with GPS data of the test location and time, which would facilitate the mapping of malaria-carrying mosquitos. If desired, the camera can be used to monitor the test in real time. In such an eventuality, the test can be terminated as soon as a detectable emission is observed.

We carried out a sequence of experiments with our chips. Each chip was used only once and disposed of after the test. In one set of experiments (n = 3), we inserted samples obtained from legs of *An. gambiae* into the chips and carried out the amplification process. The reactor containing primers specific to *An. gambiae* lit up while the other two reactors remained dark. In another set of experiments (n = 3), the samples were obtained from *An. arabiensis*. The reactor containing primers specific to *An. arabiensis* lit up while the other two reactors remained dark. In summary, in *all* the experiments, the chips distinguished correctly between *An. gambiae* and *An. arabiensis* without any false positives and false negatives.

### Gel Electrophoresis

**Figure 6:**
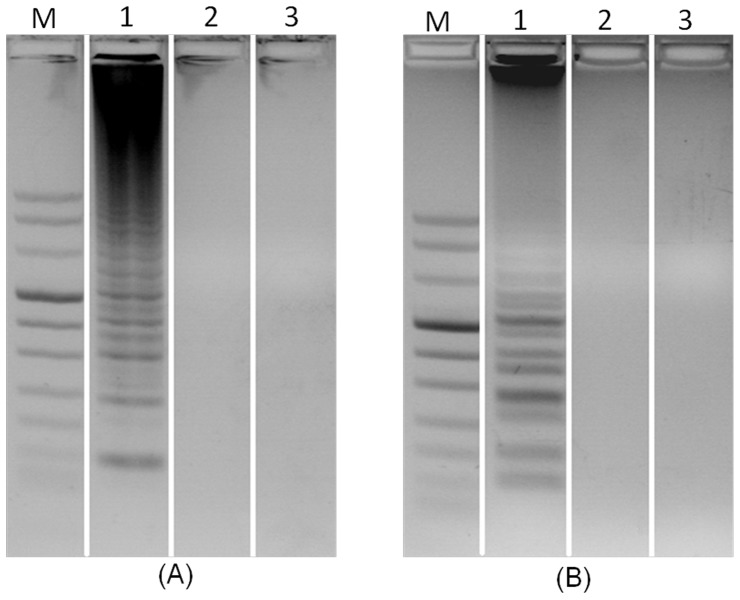
Electropherograms of LAMP products in a 2% agarose gel. Lane M is DNA marker VIII. (A) *An. gambiae* identification. Lanes 1, 2, and 3 correspond, respectively, to LAMP products from reactors G, A, and C when the sample consists of *An. Gambiae*. (B) *An. arabiensis* identification. Lanes 1, 2, and 3 correspond, respectively, to LAMP products from reactors A, G, and C when the sample consists of *An. arabiensis*.

To verify the results obtained with the intercalating dye, we subjected the amplification products to gel electrophoresis. Figure 6A and 6B are the electropherograms of ethidium bromide, stained LAMP products of samples obtained from *An. gambiae* and *An. Arabiensis,* in 2% agarose gel. In Figure 6A, lanes 1, 2, and 3 correspond, respectively, to LAMP products from reactors **G**, **A**, and **C** when the sample consists of *An. gambiae*. In contrast to PCR electropherograms that exhibit a single band, the LAMP electropherograms feature a characteristic ladder pattern [38], [41]. Consistent with the real time detection (Figure 4A) and visual results (Figure 5A), lane 1 (*An. gambiae* in a reactor **G**) features the characteristic LAMP band ladder [11] while there is no visible product in lane 2 (*An. gambiae* sample in reactor **A**) and lane 3 (negative control).

In Figure 6B, lanes 1, 2, and 3 correspond, respectively, to LAMP products from reactors **A**, **G**, and **C** when the sample consists of *An. arabiensis*. Consistent with the real time detection (Figure 4B), lane 1 (*An. arabiensis* in reactor A) features the characteristic LAMP band ladder while there is no visible product in lane 2 (*An. arabiensis* sample in G reactor) and lane 3 (negative control).

### Conclusion

In recent years, there has been a growing interest in using point-of-care diagnostics for the detection of infectious diseases and the monitoring of harmful pathogens in water and food. Here, we describe yet another, until now unexplored, application of point of care technology – gene-based species identification.

The method requires us to obtain genomic DNA from the mosquito tissue. The cells can be obtained using a whole mosquito [18] or body parts such as legs [11] or wings [38]. Here, we selected to use one of the mosquito’s legs as the source of genetic material. The presence of a gene sequence specific to a particular species is detected by carrying out isothermal amplification (LAMP) of the target sequence. Since the LAMP process is highly sensitive and specific [39]–[41], a relatively small amount of extracted DNA is sufficient for molecular-based mosquito identification.

A single-use (disposable), low-cost (estimated <$2 per test), chip that utilizes a FTA disc as a nucleic acid extraction matrix for mosquito cell capture, lysis, nucleic acid isolation, purification, and concentration was designed, constructed, and tested. The FTA disc was installed in the amplification reactor and operated in a flow-through (filtration) mode. The nucleic acids, which were captured on the FTA disc, were directly used as templates for nucleic acid amplification without a need for elution and transfer of nucleic acids, which, in turn, greatly simplified chip design and flow control.

An inexpensive, blue LED excitation light was used to excite the fluorescent dye, allowing visual detection of amplification products without a need for any expensive detection instrument. The test results were also recorded with a cell phone camera. The cell-phone can be used, among other things, for record keeping, transfer of data to a central processing facility, data analysis, and providing space and time stamps.

Future improvements of the device will include additional reactors such as a reactor for positive control, which will amplify a gene sequence common to all mosquitoes. It would also be desirable to dry store the LAMP reagents in the reactor. This can be achieved by encapsulating the dry reagents with low melting temperature paraffin as previously described elsewhere [42]. The paraffin will melt when the amplification reactor reaches the incubation temperature of 63°C. Upon melting, the reagents will get hydrated just in time to facilitate the amplification reaction. Another improvement may include storing the buffers for the isolation and purification of nucleic acid in the chip, possibly in a pouch format as previously reported elsewhere [27]. As an alternative to using a power supply or a battery for the heater, the heat needed for LAMP amplification can be supplied by an exothermic chemical reaction with paraffin providing a phase change medium to control the reactor’s temperature [23]. The incorporation of “self-heating” would result in a low-cost, completely non-instrumented, microfluidic device.

Our experiments indicate that the chip system is suitable for rapid molecular identification of malaria vector mosquitoes. The method can also be used to identify insecticide-resistant mosquitoes. Moreover, with proper modifications, we anticipate the chip can be used to detect nucleic acids associated with mosquito-borne pathogens such as malaria parasites or arboviruses, and to identify mosquito bloodmeal sources. Previously, we have used similar chips to detect infectious diseases such as HIV [31].
